# Association of sinonasal cancer incidence with occupation in the Nordic countries – elevated risk especially among woodworkers

**DOI:** 10.2340/1651-226X.2025.44875

**Published:** 2025-12-15

**Authors:** Alexandra Schindele, Lalle Hammarstedt‐Nordenvall, Antti Mäkitie, Jan Ivar Martinsen, Sanna Lappi-Heikkinen, Johnni Hansen, Elsebeth Lynge, Jenny Selander, Ingrid Sivesind Mehlum, Jóhanna Eyrun Torfadottir, Marcin W. Wojewodzic, Eero Pukkala

**Affiliations:** a Department of Clinical Sciences, Otorhinolaryngology, Umeå University, Östersund, Sweden; b Division of Ear, Nose and Throat Diseases, Department of Clinical Sciences; Intervention and Technology, Karolinska Institutet and Karolinska Hospital, Stockholm, Sweden; c Department of Otorhinolaryngology – Head and Neck Surgery, University of Helsinki and Helsinki University Hospital, Helsinki, Finland; d Research Program in Systems Oncology, Faculty of Medicine, University of Helsinki, Helsinki, Finland; e Department of Research, Cancer Registry, Norwegian Institute of Public Health, Oslo, Norway; f Finnish Cancer Registry, Institute for Statistical and Epidemiological Cancer Research, Helsinki, Finland; g Danish Cancer Institute, Danish Cancer Society, Copenhagen, Denmark; h Department of Public Health, University of Copenhagen, Denmark; i Institute of Environmental Medicine, IMM Karolinska Institutet, Stockholm, Sweden; j National Institute of Occupational Health (STAMI), Oslo, Norway; k Department of Occupational and Environmental Medicine, Copenhagen University Hospital – Bispebjerg and Frederiksberg, Copenhagen, Denmark; l Centre of Public Health Sciences, University of Iceland, Reykjavik, Iceland; m Department of Chemical Toxicology, Norwegian Institute of Public Health, Oslo, Norway; n Health Sciences Unit, Faculty of Social Sciences, Tampere University, Tampere, Finland

**Keywords:** Nose neoplasms, paranasal sinus neoplasms, head and neck neoplasms, head and neck squamous cell carcinomas, adenocarcinoma, occupational risk, incidence

## Abstract

**Background and purpose:**

The study aims to assess the occupational variation of sinonasal cancer (SNC) incidence in the Nordic population. SNC is an aggressive disease with poor prognosis and a strong connection with occupational exposure, hence, assessing occupational risk for SNC is an essential aspect in the efforts of cancer prevention.

**Patients/material and methods:**

Standardized incidence ratios (SIR) with 95% confidence intervals (CI) for SNC were calculated for 54 occupational categories from data based on population censuses and cancer registries in the five Nordic countries Denmark, Finland, Iceland, Norway, and Sweden.

**Results:**

During 1961–2005, 5,799 SNC cases were registered, 61% men and 39% women. Male woodworkers had an SIR of 1.84 for SNC (95% CI 1.66–2.04) with 355 cases, a finding consistent across all Nordic countries. The SIR for the histological subgroup sinonasal adenocarcinoma (SNAC) among male woodworkers was 5.50 (95% CI 4.56–6.56) with 122 cases. Female woodworkers also had an elevated SIR for SNC of 1.88 (95% CI 0.90–3.46), but based on only 10 cases. Country-specific elevated SIRs for SNC in men were noted in Denmark for shoe and leather workers (SIR 3.62, 95% CI 1.33–7.87), and in Norway for smelting workers (SIR 2.24, 95% CI 1.41–3.39). Reduced SIRs were observed for male military personnel, teachers, gardeners and farmers, and female religious workers.

**Interpretation:**

According to these Nordic registry data, woodworking, which is normally based on soft wood in the Nordic countries, is a high-risk occupation for SNC and particularly for SNAC.

## Introduction

Sinonasal cancer (SNC), that is, cancer of the nasal cavity and paranasal sinuses, is a rare but aggressive disease and often diagnosed at a late stage and with poor prognosis. It comprises a histologically heterogenous group of tumors that have been associated with several occupational and behavioral exposures.

According to data from 14 countries mainly in Europe, North America, and the Asia-Pacific region, SNC comprised less than 1% of all malignant tumors in 2004–2008 [1]. The age-standardized (World Standard) incidence rate in the Nordic countries in 2019–2023 was 0.6 per 100,000 person-years for men and 0.4 for women, and these rates have been relatively constant since the 1980s [2]. The 5-year age-standardized relative survival of SNC patients diagnosed over the period 2019–2023 in the Nordic countries was 60% for men and 61% for women [2].

Nasal cavity is the most frequent location of SNC tumor (71%), followed by maxillary sinus (17%) and ethmoid and frontal sinuses (2%) in the Danish population studied over a period from 1980 to 2014 [3]. In a US population studied between 1973 and 2015, the most common SNC locations were the nasal cavity (46%), maxillary (32%), and ethmoid sinus (9%) [4]. Sinonasal carcinomas are dominantly of epithelial origin. In USA during the period of 1973–2006, squamous cell carcinomas (SCC) were most common in more than 50% of cases, followed by adenocarcinomas (SNAC) arising from mucus-secreting glandular tissue (10–20% of the cases) [5]. SNAC can be further divided into intestinal-type adenocarcinoma (ITAC), and non-ITAC [6].

Occupational exposures to certain substances have been associated with an increased risk of SNC. Wood dust, the woodworking by-product in industrial sectors working with wood, affects millions of workers around the world [7]. Examples of wood industrial sectors are forestry, construction, manufacturing and services, such as carpenters. The International Agency for Research on Cancer (IARC) has classified wood dust as a Group 1 carcinogen to humans, particularly for SNC, based on many epidemiological studies [8]. Leather dust exposure, studied in epidemiological studies of boot and shoe manufacture and repair, can also result in SNC, and is considered a Group 1 carcinogen to humans according to IARC [9]. Exposure to hardwood and leather dust have a powerful and confirmed connection with SNAC [10], and for hardwood dust, particularly with ITAC [6]. An increased SNC risk has also been found strongly related to nickel exposure, especially for nickel sulphate exposure, and combinations of nickel sulphides and oxides encountered in the nickel refining industries [11, 12]. Finally, IARC has classified formaldehyde, chromium (VI) compounds, carpentry/joinery, and work in textile manufacturing industry as carcinogenic with limited evidence in humans [13, 14].

There is little evidence on occupational variation in the incidence of SNC. Our aim is to assess occupational variation of SNC incidence using the large scale data collected by the Nordic Occupational Cancer (NOCCA) project [15]. Identifying high-risk occupations may increase awareness among national occupational health and safety professionals and policymakers in cancer prevention efforts.

## Patients/material and methods

We used the data collected by the NOCCA study, based on individuals compulsory participating in population censuses in the Nordic countries Denmark, Finland, Iceland, Norway and Sweden between 1960 and 1990. The cohort comprises 14.9 million persons and includes participants aged 30–64 years in the beginning of the follow-up. The NOCCA project uses the unique personal identity codes given to all residents in each of the Nordic countries, which secure high-quality data, linking individuals to occupational and cancer registries. The follow-up of the individual started in the beginning of the year following the first available census when the person was 30–64 years old and ended at emigration, death, or country specific closing date (end of year 2003 in Denmark and Norway, 2004 in Iceland, and 2005 in Finland and Sweden).

### Occupational data

For occupational data, the national censuses collecting information on occupation and industry, are used. The original, national occupation codes are for project purpose re-classified into 53 occupational categories, and a group of economically inactive individuals. Each individual is classified based on the occupation recorded in the beginning of the follow-up.

### Cancer data

In the NOCCA project, cancer cases are collected from the cancer registry in each Nordic country, which are nationwide and population-based with recordings starting from 1943 to 1958 [16]. The completeness of data on incident cancers is very high. Data on new cancer cases are collected from public hospitals, pathology departments, primary care units, private clinics, and (except for Sweden) death certificates.

Most SNC cases were coded for topography according to the International Classification on Diseases, version 7 (ICD-7), code 160, specifically; tumor in the nasal cavity (160.0); tumor in the eustachian tube and middle ear (160.1); tumor in the maxillary sinus (160.2); tumor of other specified sinus (160.7); tumor with multiple localization (160.8); tumor without specified localization (160.9). For morphology, coding according to MOTNAC, ICD-O-1, ICD-O2, and ICD-O3 were used, depending on country and timespan [15]. The results are presented for the category of all SNC cases comprising all histological types. In addition, results for all other countries except Denmark are presented for the histological subgroup SNAC.

### Statistics

The standardized incidence ratio (SIR) describes the relative risk of SNC/SNAC incidence in each occupational category with reference to the SNC/SNAC incidence rates for the entire national populations. Thus, SIR is a ratio of the observed number of SNC/SNAC cases in each occupation and the expected number of SNC/SNAC cases, calculated by multiplying person-years created by the cohort members with the reference incidence rate, stratified by country, sex, period (5-year categories 1961–65, …, 2001–2005) and age (5-year categories 30–34, …, 85+). The exact 95% confidence interval (CI) for each SIR is defined assuming a Poisson distribution of observed number of cases.

## Results

In total, 5,799 SNC cases were registered during 1961–2005. The majority, 61%, were men (Table 1). SNAC comprised 11.5% (527 cases) out of all SNC cases in Finland, Iceland, Norway and Sweden. The SNAC subgroup consisted of 73% men and 27% women.

**Table 1:** Study population, follow-up periods, and number of sinonasal cancer (SNC) cases (total) and the histological subgroup of sinonasal adenocarcinoma (SNAC) cases in men and women stratified by country.

Country	Study population (*M*)	Follow-up period	Number of SNC cases (total)		Number of SNAC cases (subgroup)	
			Men	Women	Men	Women
Denmark	2.0	1971–2003	747	469		
Finland	3.4	1971–2005	520	396	35	25
Iceland	0.1	1982–2004	21	19	< 5	< 5
Norway	2.6	1961–2003	770	483	103	47
Sweden	6.8	1961–2005	1,465	909	246	69
Total	14.9		3,523	2,276	386	141

Data on SNAC were not available from Denmark.

Median age at diagnosis for male SNC cases was 67 years, and 9.5% of cases were represented from the youngest age group (30–49 years), 53.8% from ages 50 to 69 years, and 36.7% from 70+ years. Corresponding figures for female SNC cases were 63 years, 7.9%, 45.9%, and 46.2%.

Male SNAC cases had a median age of 65 years at diagnosis, 8.8% of cases from ages 30 to 49, 58.3% from 50 to 69 years, and 32.9% from 70+ years of age. Female SNAC cases had a median age of 68 years, with 9.9% from ages 30 to 49, 42.6% from 50 to 69 years, and 47.5% from 70+ years of age.

### SNC and occupation in men

The highest SIR for SNC in men was among woodworkers (SIR 1.84, 95% CI 1.66–2.04) (Table 2). Elevated SIRs were consistent in all Nordic countries, with 2.64 (95% CI 2.07–3.31) in Denmark, 1.46 (95% CI 1.07–1.95) in Finland, 1.94 (95% CI 0.05–10.82) in Iceland, 1.35 (95% CI 1.06–1.70) in Norway, and 2.04 (95% CI 1.75–2.38) in Sweden.

**Table 2:** Standardized incidence ratios (SIR), with 95% confidence intervals (CI), for sinonasal cancer in the Nordic countries 1961–2005, by sex and occupational category.

Occupational category	Men			Women		
	Obs	SIR	95% CI	Obs	SIR	95% CI
Technical workers	206	0.88	0.77–1.01	8	1.04	0.45–2.05
Physicians	12	0.78	0.40–1.37			
Nurses				28	0.89	0.59–1.29
Assistant nurses	6	1.49	0.55–3.25	36	0.96	0.67–1.33
Other health workers	12	0.68	0.35–1.20	18	0.76	0.46–1.18
Teachers	60	0.69	0.53–0.89	55	0.94	0.71–1.22
Religious workers	46	0.88	0.65–1.18	9	0.52	0.24–0.98
Artistic workers	12	0.74	0.38–1.30			
Journalists	6	0.80	0.29–1.73			
Administrators	164	1.02	0.87–1.19	6	0.46	0.17–1.01
Clerical workers	105	0.89	0.73–1.07	191	1.08	0.94–1.25
Sales agents	118	0.84	0.70–1.00	25	1.19	0.77–1.76
Shop workers	95	0.95	0.77–1.17	100	0.84	0.69–1.02
Farmers	341	0.81	0.72–0.90	46	0.76	0.55–1.01
Gardeners	72	0.72	0.56–0.91	62	0.99	0.76–1.28
Fishermen	56	1.33	1.00–1.72			
Forestry workers	75	1.06	0.84–1.33			
Miners and quarry workers	12	0.72	0.37–1.25			
Seamen	41	1.03	0.74–1.40			
Transport workers	56	0.93	0.70–1.21			
Drivers	184	1.11	0.96–1.28	6	1.85	0.68–4.02
Postal workers	32	0.97	0.66–1.37	29	1.11	0.75–1.60
Textile workers	38	1.15	0.82–1.58	59	0.98	0.75–1.27
Shoe and leather workers	17	1.35	0.79–2.16			
Smelting workers	66	1.20	0.93–1.53			
Mechanics	231	0.99	0.87–1.13	11	1.21	0.61–2.17
Plumbers	27	0.99	0.65–1.44			
Welders	29	1.13	0.76–1.62			
Electrical workers	79	0.95	0.76–1.19	10	1.42	0.68–2.60
Wood workers	355	1.84	1.66–2.04	10	1.88	0.90–3.46
Painters	43	0.90	0.65–1.21			
Building hands	137	1.24	1.05–1.47			
Bricklayers	29	1.00	0.67–1.43			
Printers	22	0.81	0.51–1.22	5	0.89	0.29–2.07
Chemical process workers	47	1.10	0.81–1.46	5	1.04	0.34–2.42
Food workers	47	0.83	0.61–1.10	31	1.29	0.87–1.83
Beverage workers	8	2.09	0.90–4.12			
Glass makers	50	1.13	0.84–1.49	11	1.00	0.50–1.80
Packers, loaders, warehouse workers	77	0.95	0.75–1.19	26	1.52	0.99–2.23
Engine operators	72	1.05	0.82–1.32			
Public safety workers	42	0.92	0.66–1.24			
Cooks and stewards	11	1.27	0.63–2.27	20	0.85	0.52–1.31
Domestic assistants				64	1.06	0.82–1.35
Waiters	8	1.45	0.63–2.86	20	0.84	0.52–1.30
Building caretakers	38	1.02	0.72–1.41	114	1.04	0.87–1.25
Hairdressers	8	0.98	0.42–1.94	13	1.35	0.72–2.31
Launderers	8	1.58	0.68–3.12	14	1.03	0.56–1.73
Military personnel	11	0.43	0.22–0.77			
Economically inactive	181	1.05	0.91–1.22	1,187	1.02	0.97–1.08
All categories	3,523	1.00	Reference	2276	1.00	Reference

Results based on less than five observed cases (Obs) are not reported.

In addition, there were country-specific elevated SIRs in certain occupations. In Denmark, shoe and leather workers had an elevated SIR of 3.62 (95% CI 1.33–7.87). In Finland, other construction workers (building hands) had an SIR of 1.75 (95% CI 1.22–2.42). In Norway, smelting workers had increased SIR of 2.24 (95% CI 1.41–3.39), and glass, ceramic, and tile workers had an SIR of 2.09 (95% CI 1.14–3.51).

Low SIRs in all Nordic countries combined were observed for military personnel (SIR 0.43, 95% CI 0.22–0.77), teachers (SIR 0.69, 95% CI 0.53–0.89), gardeners (SIR 0.72, 95% CI 0.56–0.91), and farmers (SIR 0.81, 95% CI 0.72–0.90). In Norway specifically, also sales agents (SIR 0.64, 95% CI 0.40–0.98) and technical workers (SIR 0.56, 95% CI 0.30–0.92) had a reduced incidence for SNC.

### SNAC and occupation in men

Woodworkers had the highest SIR for SNAC (SIR 5.50, 95% CI 4.56–6.56). The SIR in Finland was 2.85 (95% CI 1.05–6.20), in Norway 3.77 (95% CI 2.49–5.49), and in Sweden 6.83 (95% CI 5.48–8.41).

Low SIRs for SNAC in men were observed in forestry workers (SIR 0.11, 95% CI 0.00–0.60), other workers (SIR 0.22, 95% CI 0.05–0.64), clerical workers (SIR 0.43, 95% CI 0.16–0.95), farmers (SIR 0.56, 95% CI 0.35–0.84), and technical workers (SIR 0.61, 95% CI 0.36–0.98).

### SNC and occupation in women

Female woodworkers had an SIR of 1.88 (95% CI 0.90–3.46), similar as in men but based on only 10 cases (Table 2). Packers, loaders, and warehouse workers had an SIR of 1.52 (95% CI 0.99–2.23), due to elevated SIRs of 6.05 and 2.25 in Denmark and Norway, respectively.

A reduced incidence for SNC in women was observed in religious workers (SIR 0.52, 95% CI 0.24–0.98).

### SNAC and occupation in women

There was no clearly elevated or reduced incidence of SNAC in women in the Nordic countries in any of the 54 occupational categories.

### SNC and SNAC in woodworkers by time period and age group

The SIR for male woodworkers was slightly increasing between 1961 and 2005 for SNC and the histological subgroup SNAC (Table 3). The excess was mainly observed in age groups of 50–69 and 70+ years. Virtually no excess was observed in other histologies of SNC than SNAC. The number of observations for female woodworkers is too small for stratification.

**Table 3:** Numbers of sinonasal cancer cases (Obs) and standardized incidence ratios (SIR), with 95% confidence intervals, for male woodworkers by histology, time period, age, and country at diagnosis in the Nordic countries.

Category	Sinonasal cancer					
	All types (355 cases)		Adenocarcinoma^*^ (122 cases)		Other^*^ (159 cases)	
	Obs	SIR	Obs	SIR	Obs	SIR
Time period						
1961–1975	80	1.72 (1.36-2.14)	29	4.72 (3.16-6.77)	42	1.14 (0.82-1.53)
1976–1990	142	1.79 (1.50-2.10)	44	5.32 (3.87-7.14)	66	1.14 (0.88-1.45)
1991–2005	133	1.99 (1.66-2.36)	49	6.30 (4.66-8.33)	51	1.11 (0.83-1.46)
Age						
30-49	19	1.21 (0.73-1.89)	<5		14	1.17 (0.64-1.96)
50-69	195	1.94 (1.67-2.23)	72	5.81 (4.54-7.32)	74	1.03 (0.81-1.29)
70+	141	1.84 (1.55-2.17)	48	5.97 (4.40-7.92)	71	1.22 (0.96-1.54)
Country						
Denmark	74	2.66 (2.09-3.34)				
Finland	46	1.46 (1.07-1.95)	6	2.85 (1.05-6.20)	40	1.38 (1.00-1.86)
Iceland	<5		<5		<5	
Norway	74	1.35 (1.06-1.70)	27	3.77 (2.49-5.49)	47	0.98 (0.72-1.30)
Sweden	160	2.04 (1.74-2.38)	88	6.83 (5.48-8.41)	72	1.09 (0.85-1.37)

Data on subtypes of sinonasal cancer not available from Denmark.

* Excludes Denmark.

## Discussion and conclusion

We assessed occupational variation of SNC incidence using the NOCCA database, which showed an elevated SIR of 1.84 for male woodworkers. The histological subgroup SNAC had even higher SIR of 5.50 in the same occupation. Furthermore, male shoe- and leatherworkers, smelting workers, glass, ceramic, and tile workers, as well as other construction workers, had elevated SIRs in separate countries. Female woodworkers had an excess for SNC, and female packers, loaders, and warehouse workers had high SIRs in Denmark and Norway, but based on few cases. Our results are in line with an international meta-analysis which showed a relative risk of 1.61 for SNC and exposure to wood dust. Exposures to leather dust, formaldehyde, textile industry, construction, nickel, and chromium compounds showed an increased risk of SNC but to a lesser degree [17].

In the woodworking occupation, the SIR for SNAC among men in this cohort increased from 4.72 during the sixties to mid-seventies, up to 6.30 from 1991 to 2005. The SIR in the category of other histological types of SNC was consistently close to 1.0. Hence, the elevated SIR in the total SNC is almost entirely due to excess risk of SNAC. This result is in line with a previous Nordic study by Siew et al., which demonstrated a strong dose–response association between cumulative exposure to wood dust and SNAC, but no association with other types of SNC [18].

Wood dust can be derived from both hardwood and softwood, the first originating from deciduous, broad-leaved trees which are dense and slow growing, such as birch, beech, ash, alder, aspen, and oak, species more common in southern areas of the Nordic countries. Softwood comes from coniferous, needle-leaved and evergreen trees, such as pine and spruce dominating the northern parts, which develop at a quicker pace and are less dense, more flexible and lighter, making up 80% of global timber. In the Nordic countries, the majority of woodworkers are exposed to softwood [15]. In our data, SNAC corresponds to 11.5% of all SNC in the Nordic population (excluding Denmark), which is in line with a study by Leivo et al. comparing populations typically exposed to softwood (Finland), and hardwood dust (France) [6]. In Finland, the proportion of SNAC out of SNC was 9%, with a dominance of n-ITAC. In France, the SNAC proportion was 37%, predominantly ITAC. In our group of male woodworkers from Finland, Norway and Sweden, 43% of the SNC cases had SNAC, which is a low proportion as compared to a group of woodworkers in Portugal, with 85% SNAC (ITAC) out of 33 SNC cases, exposed primarily to hardwood dust [19].

People in less-developed countries are probably more at risk from occupational exposures, which highlights the importance of research in this field also in middle- and low-income countries worldwide.

Shoe and leather workers had an elevated risk of 3.62 in men in Denmark, but no significant elevated numbers for the Nordic population overall. A review on SNC and leather dust exposure shows that four out of five case-control studies, and five out of six cohort studies found an excess risk of SNC associated with leather dust exposure in shoe trades [20].

In an earlier report on occupation-specific cancer incidence based on censuses from Denmark, Finland, Norway and Sweden with follow-up from 1971 to 1991, shoe and leather workers had an SIR of 2.94 (95% CI 1.47–5.26) [21]. This might reflect increasing awareness of exposure hazards in the leather processing industry in the Nordic countries over the past years.

Nickel is a metal used in alloys with other metals to produce stainless steel, electroplating, and as cathode material in rechargeable batteries in electrical vehicles. The demand for nickel is expected to increase strongly due to its key role in the energy transition. IARC has classified nickel compounds as carcinogenic to humans (Group 1) and with sufficient evidence for SNC in humans [12]. An excess incidence of SNC has also been reported in studies of nickel refinery workers [22, 23]. In our study, an elevated SIR of 2.24 was observed for male smelting workers in Norway. The category smelting workers in the NOCCA project includes nickel process workers. A large nickel refinery located in Kristiansand since the mid-19th century might explain the noted excess risk. Andersen et al. showed an SIR of 18 (95% CI 12–25) for nasal cancer for persons who were employed before 1956 at this nickel refinery plant in Norway, with a dose–response relation for both nickel oxide (water insoluble) and soluble nickel [22]. In Finland, persons working in a nickel smelter and refinery located in Harjavalta, had an SIR of 27 for SNC among the refinery workers in a follow-up up to 2011 [21]. In 1970, changes were made in the refinery processes, and no SNC cases were observed among persons employed after that year. Also, the common use of respiratory masks started around 1985, and became mandatory from 1990 [23].

Apart from occupational exposure, few other risk factors have been identified for SNC. However, tobacco smoking and oncogenic viruses, are discussed as potential risk factors. Studies on tobacco smoking and risk for SNC show inconsistent results. According to IARC, there is sufficient evidence that smoking causes cancer in the nasal cavity and paranasal sinuses, based on case-control studies supporting the existence of causal association [24]. Studies show a significantly increased relative risk for SCC, with an OR of 1.72 among current smokers, but no association observed for SNAC [25]. In earlier Nordic papers on SNC and wood dust exposure, smoking is not considered a risk factor for SNC [26]. Since the histological subgroup SNAC explains the elevated SIRs for SNC in male woodworkers in the Nordic cohort, it is unlikely that tobacco smoking would contribute markedly to this finding.

SNC is a diverse disease with likely multiple etiologies, including carcinogenic exposures not only to irritant substances, but also to carcinogenic microbes. The role of human papilloma virus (HPV) in SNC is not established but a potential role in a subset of SNCs has been suggested [27]. According to a systematic review on HPV in SNC of 57 studies by Sjöstedt et al., around 30% of sinonasal SCCs are HPV positive, while HPV does not seem to be associated with SNAC [28]. A distinct tumor entity of sinonasal SCCs is the HPV-related multiphenotypic sinonasal carcinoma (HMSC). This subset of SNCs is associated with high-risk HPV genotypes, predominantly type 33 [29]. A Swiss study noted a potential occupational association between nursing and HMSC, with three out of four cases of HMSC found in the nasal cavity of nurses and nursing assistants with regular patient contact [30]. A systematic review concludes that surgical smoke from treatment of HPV lesions can contain HPV DNA and can contaminate the upper airways of operating room personnel [31]. However, the SIR values in this Nordic cohort were not elevated in the occupations where occupational HPV exposure might be high, such as for physicians, dentists, nurses, and other types of health workers.

### Strengths and limitations

SNC is rare, and therefore large population studies are needed to examine the risk in occupational groups. With a cohort of 14.9 million persons, and a total of 5,799 SNC cases, our study is likely the largest study on occupational SNC ever published.

SNC in the NOCCA data are based on the ICD-7 coding structure, which includes the eustachian tube and middle ear (160.1). We have no means to exclude these cases due to a preset definition in data retrieval; however, malignancies of the middle ear and eustachian tube are very uncommon, with an estimated middle ear carcinoma incidence of less than 0.02 per 100 000 person-years in the USA in 2011 [32]. Thus, the cases with eustachian tube and middle ear carcinomas will not have an important impact on the SIRs for SNC.

The NOCCA project had no data on SNAC from Denmark, due to shortcomings in histology coding in the 1970s. Furthermore, data on other histological subtypes than SNAC were not tabulated, nor was the subdivision of SNAC into ITAC/n-ITAC available from the historical Cancer Registry data.

This study is based on data sampled almost two decades ago, with the risk of missing out current occupational risk factor trends in SNC. On the other hand, a cancer disease can take decades to develop, associations seen with exposures occurring more than 20 or 30 years before the time of diagnosis [10], and data on occupational exposures are therefore relevant to study in a long timeframe.

### Study interpretation

Nordic registry data reveal woodworking to be a high-risk occupation for SNC, with elevated SIR of 1.84 for men, and explained by the histological subgroup SNAC with the SIR as high as 5.50. Women had a similar excess SIR of 1.88 for SNC in the same occupation, but based on few cases. In light of these findings, it is paramount to further study occupational risks for SNC in cancer prevention efforts, preferably in subtypes of SNC. The coming update of the NOCCA database will hopefully give new insights into this field.

## Data Availability

Most of the tabulated data are available on https://astra.cancer.fi/NOCCA/.

## References

[1] Youlden DR, Cramb SM, Peters S, Porceddu SV, Møller H, Fritschi L, et al. International comparisons of the incidence and mortality of sinonasal cancer. Cancer Epidemiol. 2013;37(6):770–9. 10.1016/j.canep.2013.09.014 24138871

[2] Larønningen S, Arvidsson G, Bray F, Dahl-Olsen E, Engholm G, Ervik M, et al. NORDCAN: cancer incidence, mortality, prevalence and survival in the Nordic Countries, Version 9.5 (19.06.2025) Association of the Nordic Cancer Registries. Cancer Registry of Norway. 2025 [cited 2025 Aug 19]. Available from: https://nordcan.iarc.fr/

[3] Sjöstedt S, Jensen DH, Jakobsen KK, Grønhøj C, Geneser C, Karnov K, et al. Incidence and survival in sinonasal carcinoma: a Danish population-based, nationwide study from 1980 to 2014. Acta Oncol. 2018;57(9):1152–8. 10.1080/0284186x.2018.1454603 29578367

[4] Gore MR. Survival in sinonasal and middle ear malignancies: a population-based study using the SEER 1973–2015 database. J BMC Ear Nose Throat Disord. 2018;18:13. 10.1186/s12901-018-0061-4 PMC6085622 30116158

[5] Turner JH, Reh DD. Incidence and survival in patients with sinonasal cancer: a historical analysis of population-based data. Head Neck. 2012;34(6):877–85. 10.1002/hed.21830 22127982

[6] Leivo I, Holmila R, Luce D, Steiniche T, Dictor M, Heikkilä P, et al. Occurrence of sinonasal intestinal-type adenocarcinoma and non-intestinal-type adenocarcinoma in two countries with different patterns of wood dust exposure. Cancers (Basel). 2021;13(20):1-11. 10.3390/cancers13205245 PMC8533857 34680393

[7] Mofidi A, Tompa E, Kalcevich C, McLeod C, Lebeau M, Song C, et al. Occupational exposure to wood dust and the burden of nasopharynx and sinonasal cancer in Canada. Int J Environ Res Public Health. 2022;19(3):1-16. 10.3390/ijerph19031144 PMC8834578 35162168

[8] IARC Working Group. Wood dust and formaldehyde. IARC monographs on the evaluation of the carcinogenic risks to humans. Volume 62. Lyon, France: International Agency for Research on Cancer; 1995.

[9] Straif K, Benbrahim-Tallaa L, Baan R, Grosse Y, Secretan B, El Ghissassi F, et al. A review of human carcinogens – part C: metals, arsenic, dusts, and fibres. Lancet Oncol. 2009;10(5):453–4. 10.1016/s1470-2045(09)70134-2 19418618

[10] Hernberg S, Westerholm P, Schultz-Larsen K, Degerth R, Kuosma E, Englund A, et al. Nasal and sinonasal cancer. Connection with occupational exposures in Denmark, Finland and Sweden. Scand J Work Environ Health. 1983;9(4):315–26. 10.5271/sjweh.2405 6635610

[11] International Committe on Nickel Carcinogenisis in Man. Report of the International Committee on Nickel Carcinogenesis in Man. Scand J Work Environ Health. 1990;16(1 Spec No):1–82. 10.5271/sjweh.1813 2185539

[12] IARC Working Group. Chromium, nickel and welding. IARC monographs on the evaluation of carcinogenic risks to humans. Volume 49. Lyon, France: International Agency for Research on Cancer; 1990.

[13] IARC Working Groups. Chemical agents and related occupations. IARC monographs on the evaluation of carcinogenic risks to humans. Volume 100F. Lyon, France: International Agency for Research on Cancer; 2012.

[14] IARC. List of classifications by cancer sites with sufficient or limited evidence in humans, IARC Monographs Volumes 1–139a [Internet]. Lyon, France: International Agency for Research on Cancer; 2025 [updated 2025 Oct 24; cited 2025 Nov 13]. Available from: https://monographs.iarc.who.int/wp-content/uploads/2019/07/Classifications_by_cancer_site.pdf

[15] Pukkala E, Martinsen JI, Lynge E, Gunnarsdottir HK, Sparén P, Tryggvadottir L, et al. Occupation and cancer – follow-up of 15 million people in five Nordic countries. Acta Oncol. 2009;48(5):646–790. 10.1080/02841860902913546 19925375

[16] Pukkala E, Engholm G, Højsgaard Schmidt LK, Storm H, Khan S, Lambe M, et al. Nordic Cancer Registries – an overview of their procedures and data comparability. Acta Oncol. 2018;57(4):440–55. 10.1080/0284186x.2017.1407039 29226751

[17] Binazzi A, Ferrante P, Marinaccio A. Occupational exposure and sinonasal cancer: a systematic review and meta-analysis. BMC Cancer. 2015;15:49. 10.1186/s12885-015-1042-2 25885319 PMC4339645

[18] Siew SS, Martinsen JI, Kjaerheim K, Sparén P, Tryggvadottir L, Weiderpass E, et al. Occupational exposure to wood dust and risk of nasal and nasopharyngeal cancer: a case-control study among men in four nordic countries-With an emphasis on nasal adenocarcinoma. Int J Cancer. 2017;141(12):2430–6. 10.1002/ijc.31015 28840594

[19] Teixeira-Marques F, Pacheco I, Pinheiro-Guedes L, Estêvão R, Lousan N. Sinonasal intestinal-type adenocarcinoma in northern Portugal: a woodworker’s occupational hazard. Occup Med (Lond). 2024;74(8):596–600. 10.1093/occmed/kqae085 39298678

[20] Bonneterre V, Deschamps E, Persoons R, Bernardet C, Liaudy S, Maitre A, et al. Sino-nasal cancer and exposure to leather dust. Occup Med (Lond). 2007;57(6):438–43. 10.1093/occmed/kqm050 17591601

[21] Andersen A, Barlow L, Engeland A, Kjaerheim K, Lynge E, Pukkala E. Work-related cancer in the Nordic countries. Scand J Work Environ Health. 1999;25 Suppl 2:1–116. 10507118

[22] Andersen A, Berge SR, Engeland A, Norseth T. Exposure to nickel compounds and smoking in relation to incidence of lung and nasal cancer among nickel refinery workers. Occup Environ Med. 1996;53(10):708–13. 10.1136/oem.53.10.708 8943837 PMC1128579

[23] Pavela M, Uitti J, Pukkala E. Cancer incidence among copper smelting and nickel refining workers in Finland. Am J Ind Med. 2017;60(1):87–95. 10.1002/ajim.22662 27747921

[24] IARC Working Group. Tobacco smoke and involuntary smoking. IARC monographs on the evaluation of carcinogenic risks to humans. Volume 83. Lyon, France: International Agency for Research on Cancer; 2004. PMC4781536 15285078

[25] Mannetje A, Kogevinas M, Luce D, Demers PA, Bégin D, Bolm-Audorff U, et al. Sinonasal cancer, occupation, and tobacco smoking in European women and men. Am J Ind Med. 1999;36(1):101–7. 10.1002/(sici)1097-0274(199907)36:1<101::aid-ajim14>3.0.co;2-a 10361593

[26] Kjaerheim K, Haldorsen T, Lynge E, Martinsen JI, Pukkala E, Weiderpass E, et al. Variation in Nordic work-related cancer risks after adjustment for alcohol and tobacco. Int J Environ Res Public Health. 2018;15(12): 1-23. 10.3390/ijerph15122760 PMC6313809 30563223

[27] Elgart K, Faden DL. Sinonasal squamous cell carcinoma: etiology, pathogenesis, and the role of human papilloma virus. J Curr Otorhinolaryngol Rep. 2020;8(2):111–9. 10.1007/s40136-020-00279-6 PMC7314379 32582473

[28] Sjöstedt S, von Buchwald C, Agander TK, Aanaes K. Impact of human papillomavirus in sinonasal cancer-a systematic review. Acta Oncol. 2021;60(9):1175–91. 10.1080/0284186x.2021.1950922 34319844

[29] Bishop JA, Andreasen S, Hang JF, Bullock MJ, Chen TY, Franchi A, et al. HPV-related multiphenotypic sinonasal carcinoma: an expanded series of 49 cases of the tumor formerly known as HPV-related carcinoma with adenoid cystic carcinoma-like features. Am J Surg Pathol. 2017;41(12):1690–701. 10.1097/pas.0000000000000944 28877065 PMC5680105

[30] Rupp NJ, Camenisch U, Seidl K, Rushing EJ, Anderegg N, Broglie MA, et al. HPV-related multiphenotypic sinonasal carcinoma: four cases that expand the morpho-molecular spectrum and include occupational data. Head Neck Pathol. 2020;14(3):623–9. 10.1007/s12105-019-01079-1 31571045 PMC7413931

[31] Fox-Lewis A, Allum C, Vokes D, Roberts S. Human papillomavirus and surgical smoke: a systematic review. Occup Environ Med. 2020;77(12):809–17. 10.1136/oemed-2019-106333 32385189

[32] Feng L, Jin A, Dai B, Li Y, Guo Y, Wang D, et al. Outcomes of 18 cases with squamous cell carcinoma of middle ear who underwent both surgery and post-operative radiotherapy. Acta Otolaryngol. 2016;136(2): 141–3. 10.3109/00016489.2015.1094825 26472473

